# The genome sequence of an erect bryozoan, *Bugulina stolonifera* (Ryland, 1960)

**DOI:** 10.12688/wellcomeopenres.18775.1

**Published:** 2023-01-18

**Authors:** Christine Wood, John Bishop, Patrick Adkins, Helen Jenkins, Marine Biological Association Genome Acquisition Lab, Wellcome Sanger Institute Tree of Life programme, Wellcome Sanger Institute Scientific Operations: DNA Pipelines collective, Tree of Life Core Informatics collective, Darwin Tree of Life Consortium

**Affiliations:** 1 Marine Biological Association, Plymouth, UK

**Keywords:** Bugulina stolonifera, an erect bryozoan, genome sequence, chromosomal, Cheilostomatida

## Abstract

We present a genome assembly from a *Bugulina stolonifera* colony (an erect bryozoan; Bryozoa; Gymnolaemata; Cheilostomatida; Bugulidae). The genome sequence is 235 megabases in span. Most of the assembly (99.85%) is scaffolded into 11 chromosomal pseudomolecules. The mitochondrial genome was also assembled and is 14.4 kilobases long.

## Species taxonomy

Eukaryota; Metazoa; Spiralia; Lophotrochozoa; Bryozoa; Gymnolaemata; Cheilostomatida; Flustrina; Buguloidea; Bugulidae; *Bugulina*; *Bugulina stolonifera* (Ryland, 1960) (NCBI:txid192920)

## Background

*Bugulina stolonifera* (formerly known as *Bugula stolonifera* – see ( Fehlauer-Ale *et al.*, 2015) is characteristically found in artificial habitats such as harbours and marinas. Although described in 1960 from South Wales (UK), the species is considered native to the NW Atlantic ( McCann *et al.*, 2019). It is widely introduced as a fouling species in the Pacific and SW Atlantic, in Europe (including the Mediterranean), and in Australia and New Zealand ( Fofonoff *et al.*, 2018).

This lightly calcified bryozoan grows upwards into a flexible bush-like colony up to 40 mm tall, with narrow branches of just two rows of zooids side-by-side. Rhizoids (stolons) growing out across the substrate from the colony base can bud to give rise to satellite upright colonies. Sexual reproduction involves the brooding of embryos that are released as non-feeding (lecithotrophic) ciliated larvae.

As with many bryozoans, the zooids (the clonal modular individuals of the colony) occur in a variety of morphologies with different particular functions in the life of the colony. In *Bugulina*, these polymorphs include feeding zooids (autozooids), putatively defensive, non-feeding zooids (avicularia) and zooids attaching the colony to the substrate (rhizoids, a type of kenozooid). *B. stolonifera* can be cultured in the lab, which has allowed comparison of the transcriptomes of these different types of zooid to identify genes that are differentially expressed during the budding and maintenance of the various polymorphs ( Treibergs & Giribet, 2020). Over 1000 genes were identified that were expressed differentially between the autozooids and the avicularia.

## Genome sequence report

**Figure 1.**
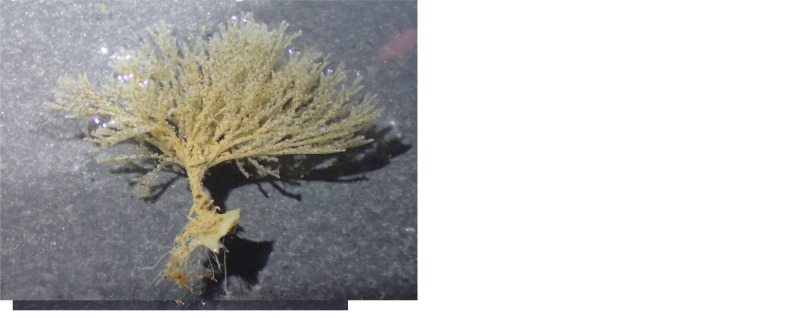
Photographs of the *Bugulina stolonifera*, tzBugStol2.1: specimen used for genome sequencing.

The genome was sequenced from a specimen of *B. stolonifera* ( Figure 1) collected from Queen Anne's Battery Marina visitors' pontoon in Plymouth (latitude 50.36, longitude –4.13). A total of 52-fold coverage in Pacific Biosciences single-molecule HiFi long reads and 99-fold coverage in 10X Genomics read clouds were generated. Primary assembly contigs were scaffolded with chromosome conformation Hi-C data. Manual assembly curation corrected 17 missing/misjoins and removed four haplotypic duplications, reducing the assembly length by 2.34% and the scaffold number by 16.67%, and increasing the scaffold N50 by 3.71%.

**Figure 2.**
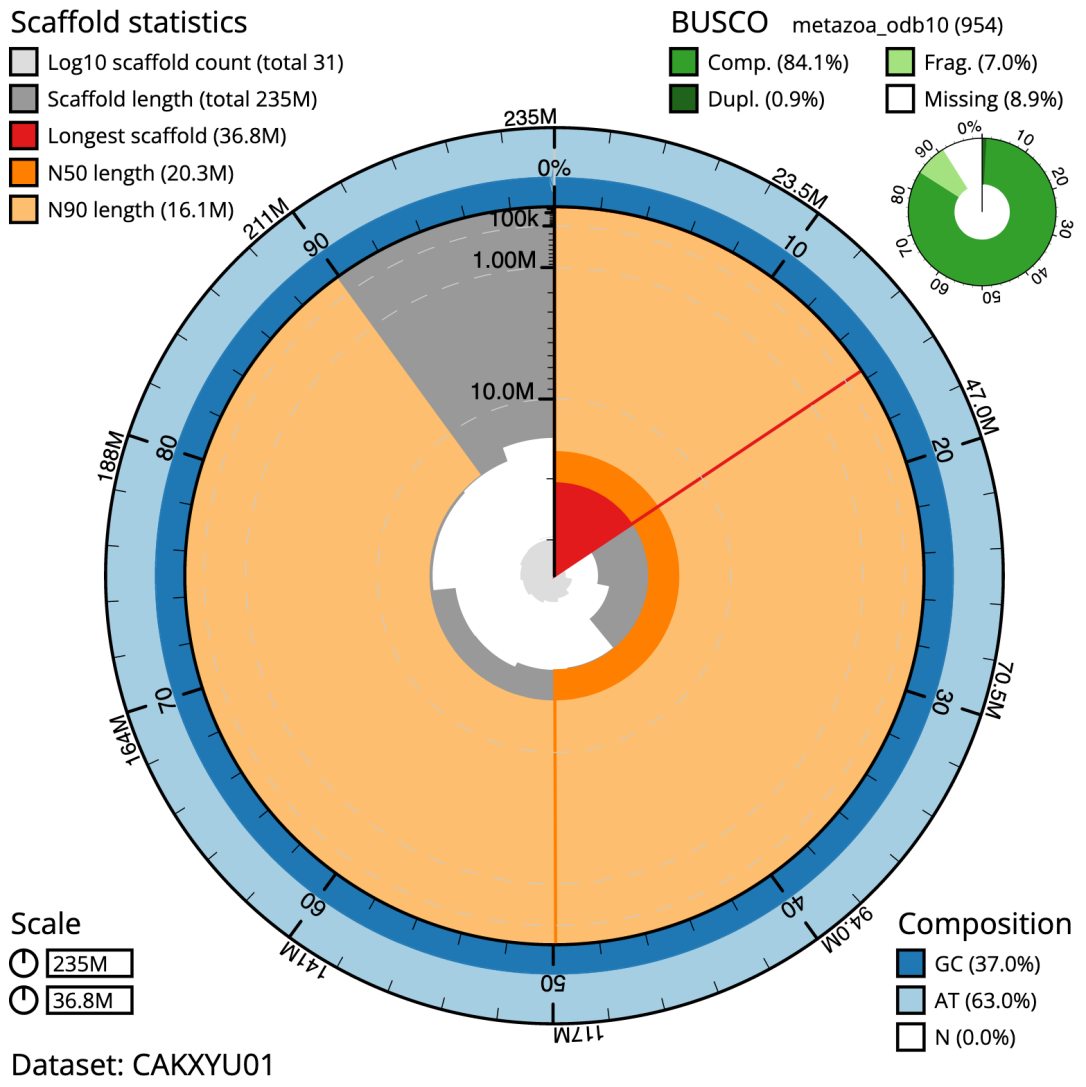
Genome assembly of *Bugulina stolonifera*, tzBugStol2.1: metrics. The BlobToolKit Snailplot shows N50 metrics and BUSCO gene completeness. The main plot is divided into 1,000 size-ordered bins around the circumference with each bin representing 0.1% of the 234,982,697 bp assembly. The distribution of scaffold lengths is shown in dark grey with the plot radius scaled to the longest scaffold present in the assembly (36,811,656 bp, shown in red). Orange and pale-orange arcs show the N50 and N90 scaffold lengths (20,271,823 and 16,134,010 bp), respectively. The pale grey spiral shows the cumulative chromosome count on a log scale with white scale lines showing successive orders of magnitude. The blue and pale-blue area around the outside of the plot shows the distribution of GC, AT and N percentages in the same bins as the inner plot. A summary of complete, fragmented, duplicated and missing BUSCO genes in the metazoa_odb10 set is shown in the top right. An interactive version of this figure is available at https://blobtoolkit.genomehubs.org/view/tzBugStol2.1/dataset/CAKXYU01/snail.

The final assembly has a total length of 235 Mb in 30 sequence scaffolds with a scaffold N50 of 20 Mb ( Table 1). Most (99.85%) of the assembly sequence was assigned to 11 chromosomal-level scaffolds ( Figure 2– Figure 5; Table 2). Chromosome-scale scaffolds confirmed by the Hi-C data are named in order of size. A heterozygous inversion was observed on chromosome 5 (7.9–9.1 Mb). While not fully phased, the assembly deposited is of one haplotype. Contigs corresponding to the second haplotype have also been deposited.

**Table 1.** Genome data for *Bugulina stolonifera*, tzBugStol2.1.

Project accession data	
Assembly identifier	tzBugStol2.1
Species	*Bugulina stolonifera*
Specimen	tzBugStol2
NCBI taxonomy ID	192920
BioProject	PRJEB51034
BioSample ID	SAMEA7536683
Isolate information	modular colony
Assembly metrics *	
Consensus quality (QV)	55.5 (Benchmark: ≥50)
*k*-mer completeness	99.99% (Benchmark: ≥95%)
BUSCO **	C:84.1%[S:83.1%,D:0.9%],F:7.0%,M:8. 9%,n:954 (Benchmark: C ≥ 95%)
Percentage of assembly mapped to chromosomes	99.85% (Benchmark: ≥95%)
Organelles	Mitochondrial genome assembled (Benchmark: complete single alleles)
Raw data accessions	
PacificBiosciences SEQUEL II	ERR8978458
10X Genomics Illumina	ERR8702816–ERR8702819
Hi-C Illumina	ERR8702820, ERR8702821
PolyA RNA-Seq Illumina	ERR10123683
Genome assembly	
Assembly accession	GCA_935421135.1
*Accession of alternate* * haplotype*	GCA_935421085.1
Span (Mb)	235.0
Number of contigs	60
Contig N50 length (Mb)	12.6
Number of scaffolds	30
Scaffold N50 length (Mb)	20.3
Longest scaffold (Mb)	36.8

* Assembly metric benchmarks are adapted from column VGP-2020 of “Table 1: Proposed standards and metrics for defining genome assembly quality” from ( Rhie *et al.*, 2021). ** BUSCO scores based on the metazoa_odb10 BUSCO set using 5.3.2. C = complete [S = single copy, D = duplicated], F = fragmented, M = missing, n = number of orthologues in comparison. A full set of BUSCO scores is available at https://blobtoolkit.genomehubs.org/view/tzBugStol2.1/dataset/CAKXYU01/busco.

**Figure 3.**
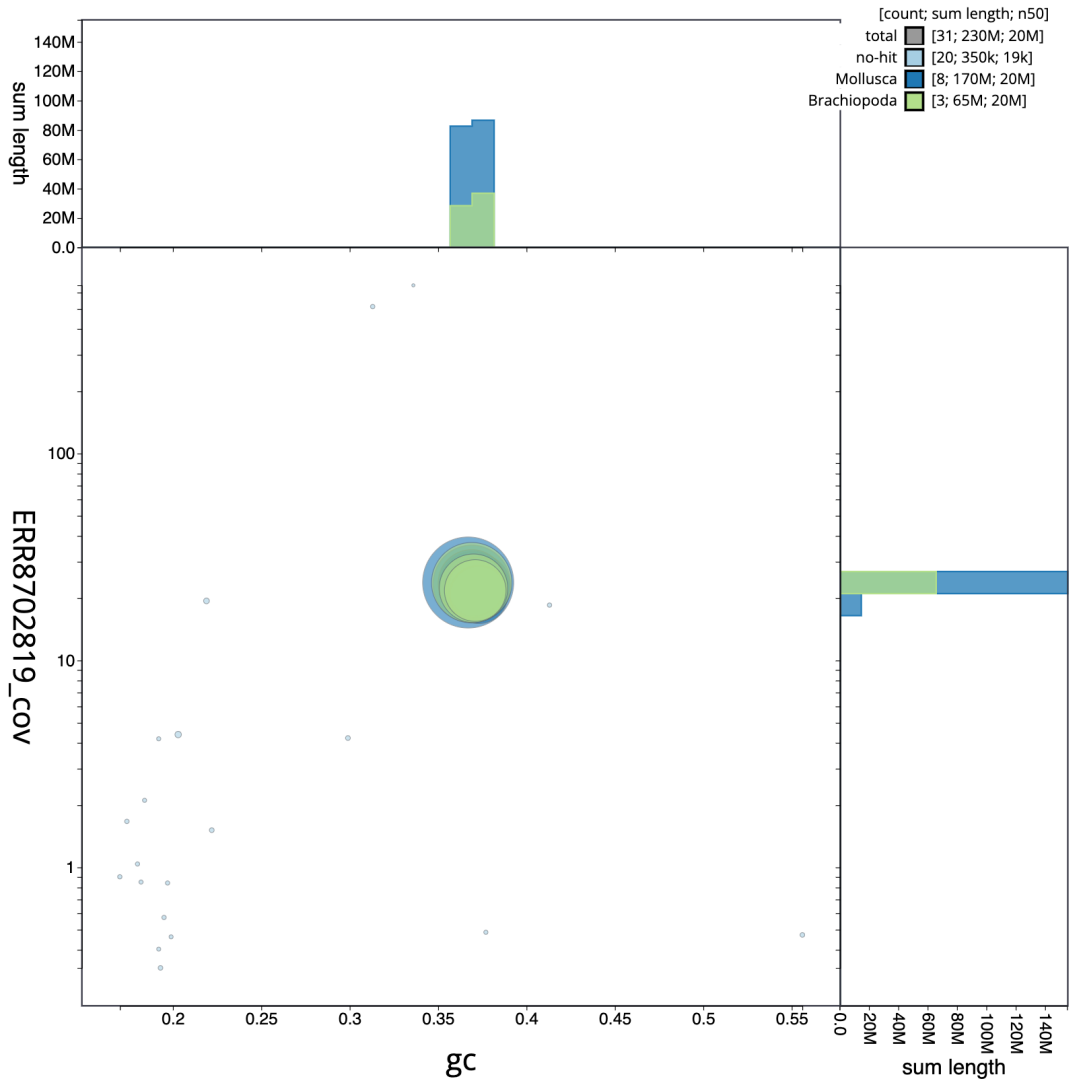
Genome assembly of *Bugulina stolonifera*, tzBugStol2.1: GC coverage. BlobToolKit GC-coverage plot. Scaffolds are coloured by phylum. Circles are sized in proportion to scaffold length. Histograms show the distribution of scaffold length sum along each axis. An interactive version of this figure is available at https://blobtoolkit.genomehubs.org/view/tzBugStol2.1/dataset/CAKXYU01/blob.

**Figure 4.**
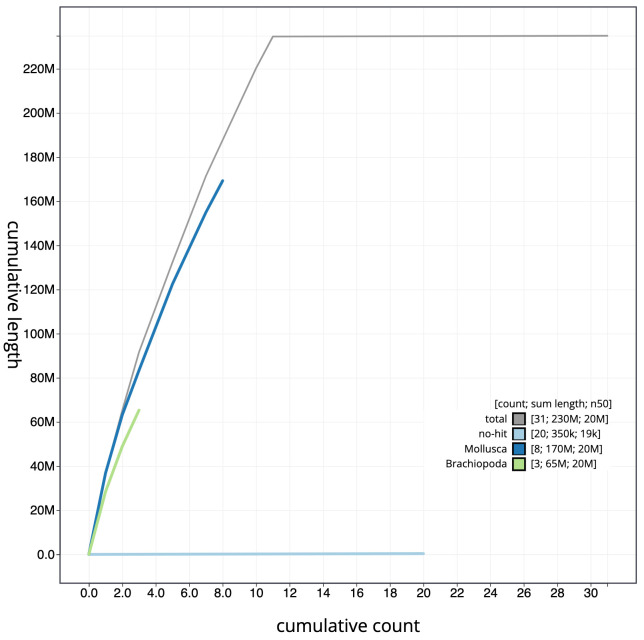
Genome assembly of *Bugulina stolonifera*, tzBugStol2.1: cumulative sequence. BlobToolKit cumulative sequence plot. The grey line shows cumulative length for all scaffolds. Coloured lines show cumulative lengths of scaffolds assigned to each phylum using the buscogenes taxrule. An interactive version of this figure is available at https://blobtoolkit.genomehubs.org/view/tzBugStol2.1/dataset/CAKXYU01/cumulative.

**Figure 5.**
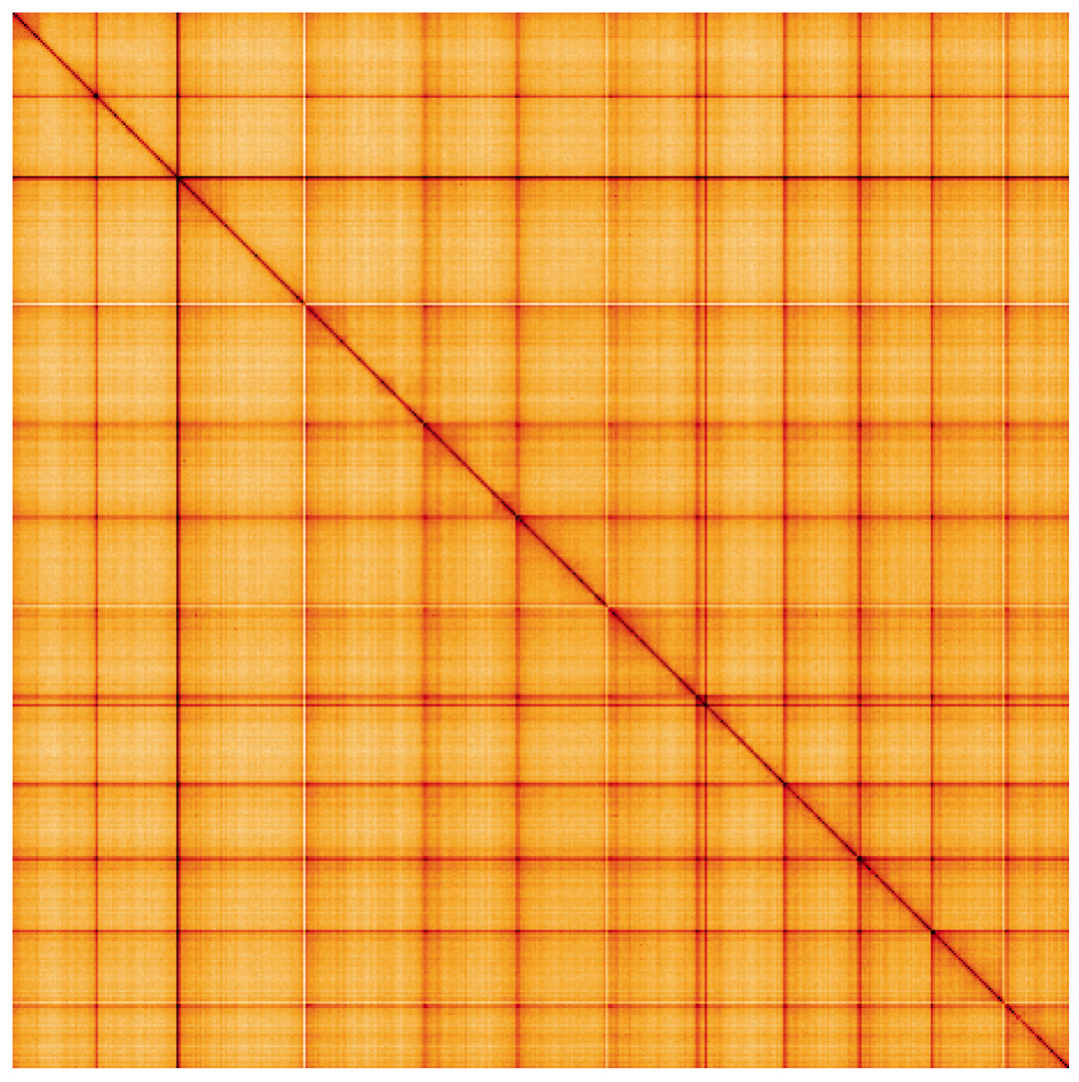
Genome assembly of *Bugulina stolonifera*, tzBugStol2.1: Hi-C contact map. Hi-C contact map of the tzBugStol2.1 assembly, visualised using HiGlass. Chromosomes are shown in order of size from left to right and top to bottom. An interactive version of this figure may be viewed at https://genome-note-higlass.tol.sanger.ac.uk/l/?d=CIFsB67eToKgl12ypgaeOw.

**Table 2.** Chromosomal pseudomolecules in the genome assembly of *Bugulina stolonifera*, tzBugStol2.

INSDC accession	Chromosome	Size (Mb)	GC%
OW285187.1	1	36.81	36.7
OW285188.1	2	28.45	36.9
OW285189.1	3	26.37	36.9
OW285190.1	4	20.48	37
OW285191.1	5	20.27	37
OW285192.1	6	19.55	37.2
OW285193.1	7	19.49	36.9
OW285194.1	8	16.4	37.1
OW285195.1	9	16.36	37.2
OW285196.1	10	16.13	37.3
OW285197.1	11	14.31	37.3
OW285198.1	MT	0.01	31.1

The assembly has a BUSCO v5.3.2 ( Manni *et al.*, 2021) completeness of 84.1% (single 83.1% and duplicated 0.9%) using the OrthoDB-v10 metazoa reference set. Although BUSCO coverage falls below the benchmark of 95%, the assembly is validated by high *k* mer coverage and consensus quality QV scores ( Table 1).

## Methods

### Sample acquisition and nucleic acid extraction

A colony of *B. stolonifera* (tzBugStol2) was collected from Queen Anne’s Battery Marina visitors’ pontoon, Plymouth, Devon, UK (latitude 50.36, longitude –4.13). The specimen was collected by hand from submerged rope on the marina pontoon and then preserved in liquid nitrogen. The collectors were Christine Wood, John Bishop and Patrick Adkins (Marine Biological Association). The specimen was identified by Christine Wood, John Bishop and Helen Jenkins (Marine Biological Association) based on its gross morphology.

DNA was extracted at the Tree of Life laboratory, Wellcome Sanger Institute (WSI). The tzBugStol2 sample was weighed and dissected on dry ice with tissue set aside for Hi-C sequencing. The tissue was disrupted using a Nippi Powermasher fitted with a BioMasher pestle. High molecular weight (HMW) DNA was extracted using the Qiagen MagAttract HMW DNA extraction kit. Low molecular weight DNA was removed from a 20 ng aliquot of extracted DNA using 0.8X AMpure XP purification kit prior to 10X Chromium sequencing; a minimum of 50 ng DNA was submitted for 10X sequencing. HMW DNA was sheared into an average fragment size of 12–20 kb in a Megaruptor 3 system with speed setting 30. Sheared DNA was purified by solid-phase reversible immobilisation using AMPure PB beads with a 1.8X ratio of beads to sample to remove the shorter fragments and concentrate the DNA sample. The concentration of the sheared and purified DNA was assessed using a Nanodrop spectrophotometer and Qubit Fluorometer and Qubit dsDNA High Sensitivity Assay kit. Fragment size distribution was evaluated by running the sample on the FemtoPulse system.

RNA was extracted from the tissue from the same specimen tzBugStol2 sample in the Tree of Life Laboratory at the WSI using TRIzol, according to the manufacturer’s instructions. RNA was then eluted in 50 μl RNAse-free water and its concentration was assessed using a Nanodrop spectrophotometer and Qubit Fluorometer using the Qubit RNA Broad-Range (BR) Assay kit. Analysis of the integrity of the RNA was done using Agilent RNA 6000 Pico Kit and Eukaryotic Total RNA assay.

### Sequencing

Pacific Biosciences HiFi circular consensus and 10X Genomics read cloud DNA sequencing libraries were constructed according to the manufacturers’ instructions. Poly(A) RNA-Seq libraries were constructed using the NEB Ultra II RNA Library Prep kit. DNA and RNA sequencing was performed by the Scientific Operations core at the WSI on Pacific Biosciences SEQUEL II (HiFi), Illumina NovaSeq 6000 (RNA-Seq and 10X) instruments. Hi-C data were also generated from tzBugStol2 using the Arima v2 kit and sequenced on the Illumina NovaSeq 6000 instrument.

### Genome assembly

Assembly was carried out with Hifiasm ( Cheng *et al.*, 2021) and haplotypic duplication was identified and removed with purge_dups ( Guan *et al.*, 2020). One round of polishing was performed by aligning 10X Genomics read data to the assembly with longranger align, calling variants with freebayes ( Garrison & Marth, 2012). The assembly was then scaffolded with Hi-C data ( Rao *et al.*, 2014) using YaHS ( Zhou *et al.*, 2022). The assembly was checked for contamination as described previously ( Howe *et al.*, 2021). Manual curation was performed using HiGlass ( Kerpedjiev *et al.*, 2018) and Pretext ( Harry, 2022). The mitochondrial genome was assembled using MitoHiFi ( Uliano-Silva *et al.*, 2021), which performed annotation using MitoFinder ( Allio *et al.*, 2020). The genome was analysed and BUSCO scores generated within the BlobToolKit environment ( Challis *et al.*, 2020). Table 3 contains a list of all software tool versions used, where appropriate.

**Table 3.** Software tools and versions used.

Software tool	Version	Source
Blobtoolkit	3.3.4	( Challis *et al.*, 2020)
freebayes	1.3.1-17- gaa2ace8	( Garrison & Marth, 2012)
Hifiasm	0.15.3	( Cheng *et al.*, 2021)
HiGlass	1.11.6	( Kerpedjiev *et al.*, 2018)
Long Ranger ALIGN	2.2.2	https://support.10xgenomics.com/ genome-exome/software/pipelines/ latest/advanced/other-pipelines
MitoHiFi	2.0	( Uliano-Silva *et al.*, 2021)
PretextView	0.2	( Harry, 2022)
purge_dups	1.2.3	( Guan *et al.*, 2020)
YaHS	1	( Zhou *et al.*, 2022)

### Ethics/compliance issues

The materials that have contributed to this genome note have been supplied by a Darwin Tree of Life Partner. The submission of materials by a Darwin Tree of Life Partner is subject to the Darwin Tree of Life Project Sampling Code of Practice. By agreeing with and signing up to the Sampling Code of Practice, the Darwin Tree of Life Partner agrees they will meet the legal and ethical requirements and standards set out within this document in respect of all samples acquired for, and supplied to, the Darwin Tree of Life Project. Each transfer of samples is further undertaken according to a Research Collaboration Agreement or Material Transfer Agreement entered into by the Darwin Tree of Life Partner, Genome Research Limited (operating as the Wellcome Sanger Institute), and in some circumstances other Darwin Tree of Life collaborators.

## Data Availability

European Nucleotide Archive: *Bugulina stolonifera* (an erect bryozoan). Accession number PRJEB51034; https://identifiers.org/ena.embl/PRJEB51034 ( Wellcome Sanger Institute, 2022). The genome sequence is released openly for reuse. The *Bugulina stolonifera* genome sequencing initiative is part of the Darwin Tree of Life (DToL) project. All raw sequence data and the assembly have been deposited in INSDC databases: The genome will be annotated using available RNA-Seq data and presented through the Ensembl pipeline at the European Bioinformatics Institute. Raw data and assembly accession identifiers are reported in Table 1.

## Author information

Members of the Marine Biological Association Genome Acquisition Lab are listed here: https://doi.org/10.5281/zenodo.4783604.

Members of the Darwin Tree of Life Barcoding collective are listed here: https://doi.org/10.5281/zenodo.4893703.

Members of the Wellcome Sanger Institute Tree of Life programme are listed here: https://doi.org/10.5281/zenodo.4783585.

Members of Wellcome Sanger Institute Scientific Operations: DNA Pipelines collective are listed here: https://doi.org/10.5281/zenodo.4790455.

Members of the Tree of Life Core Informatics collective are listed here: https://doi.org/10.5281/zenodo.5013541.

Members of the Darwin Tree of Life Consortium are listed here: https://doi.org/10.5281/zenodo.4783558.
