# Supplementary material for: The genome sequence of an erect bryozoan, Bugulina stolonifera (Ryland, 1960)
Source: Wellcome Open Res. 2023 Jan 18;8:26. [Version 1] doi: 10.12688/wellcomeopenres.18775.1 (PMC10213821; doi:10.12688/wellcomeopenres.18775.1)
Supplement: Peer review history

# Peer review: Reviewer response for version 1

**DOI:** 10.21956/wellcomeopenres.20819.r57036

**Published:** 2023-05-25

**Authors:** Marco Gerdol

**Affiliations:** 1 Department of Life Sciences, University of Trieste, Trieste, Italy

This manuscript by Wood and colleagues report the assembly of the genome of the bryozoan *Bugulina stolonifera*. This resource was obtained using gold-standard, well-established protocols and I have no major concerns concerning the process. I just have a few minor suggestions for slight improvements.

 In the background, the authors could briefly mention that genomic resources for neglected lophotrochozoan phyla, such as Bryozoa, are limited, providing a more complete rationale for generating this dataset.

 Does the assignment to 11 pseudomolecules matches with previous information about the karyotype of this species (if available)? Was the size of the assembly consistent with c-value estimates (if available)?

 “Although BUSCO coverage falls below the benchmark of 95%” -> I think the authors could add a brief comment, stating that Bryozoa have not been included in the generation of the orthoDB reference and that, more in general, Lophotrochozoa are under-represented in terms of abundance of genomic data compared with other metazoan taxa. Consequently, those missing BUSCOs may as well indicate real events of lineage-specific gene loss.

 I guess RNA-seq data was generated with an Illumina platform using a paired-end sequencing strategy. This should be specified, as the text currently does not clarify whether long reads were used for transcriptome sequencing as well.

 I think a useful data that could be easily provided and added to the text is a k-mer based estimate of the heterozygosity rate, due to its implications on the assembly of the alternate haplotype.

Are sufficient details of methods and materials provided to allow replication by others?

Yes

Is the rationale for creating the dataset(s) clearly described?

Yes

Are the datasets clearly presented in a useable and accessible format?

Yes

Are the protocols appropriate and is the work technically sound?

Yes

Reviewer Expertise:

Genomics

I confirm that I have read this submission and believe that I have an appropriate level of expertise to confirm that it is of an acceptable scientific standard.

# Peer review: Reviewer response for version 1

**DOI:** 10.21956/wellcomeopenres.20819.r57031

**Published:** 2023-05-09

**Authors:** Z. B. Randolph Quek

**Affiliations:** 1 DMERI, DSO National Laboratories, Singapore city, Singapore

In this study, Wood *et al.* sequenced and assembled the genome of a bryozoan *Bugulina stolonifera *using multiple sequencing platforms: PacBio, 10X genomics and Illumina (including HiC sequencing). They obtained a fairly comprehensive genome spanning 235 Mb over 30 scaffolds, with 11 chromosomes recovered, and a BUSCO completeness of approximately 85%. This is a step forward in the severely understudied bryozoans, and will serve as a useful genomic resource for future studies. Nevertheless, here are some (minor) suggestions I have to improve the manuscript:

1. There is little information on transcriptome assemblies and verification. Furthermore, annotation of the genome is lacking, beyond that of BUSCO completeness. While I note under "Data availability" that the authors mentioned, and I quote, "The genome will be annotated using available RNA-Seq data and presented through the Ensembl pipeline at the European Bioinformatics Institute.", it is difficult to judge if the genome is properly assembled and is limited in usefulness without annotation information. Even a preliminary *in silico *annotation might be helpful.
2. The introduction could cover some genomes of bryozoans sequenced to date, and why it is important to sequence bryozoan genomes. I note that there is a statement at the end citing Treibergs & Giribet, but more information would be useful. For example, what are some of the currently available genomes and how complete are they? What can the genomes be used for (e.g. hybrid capture design, DGE in evo-devo)? Because this is a genome report, there is no "Discussion" *per se *and it would be useful to have some information in the introduction. 
  I presume *B. stolonifera *can be a model organism for bryozoan studies, and this genome would be a valuable contribution?
3. I would like more information about the removal of sequences, if any, for contamination. How many sequences were removed, and how were they checked to be putatively non-bryozoan? A reference to Howe *et al.* (2021) is insufficient information for readers to determine if potential bryozoan contigs were indeed removed, considering their early origin in the ToL.
4. Was the mitochondrial genome complete? Are all PCGs, tRNAs and rRNAs recovered? Is 14.4 kb an expected and reasonable length?
5. I would recommend that the completeness of the genome be specified in the abstract so as to better inform readers to the quality of the genome recovered.
6. Are there any plans to improve the quality of the genome eventually? 

Other comments:

- Ideally, keywords should not overlap with words present in the title to increase visibility.
- I appreciate the uploading of raw data, but if the transcriptome data is to be used in a separate update, it might be prudent to keep access to it limited for now.

Overall, I think this is a commendable study and a step forward in understanding the biology and evolution of bryozoans. The comments made are minor and I think the authors have sufficiently specified the limitations of their assembly.

Are sufficient details of methods and materials provided to allow replication by others?

Yes

Is the rationale for creating the dataset(s) clearly described?

Partly

Are the datasets clearly presented in a useable and accessible format?

Yes

Are the protocols appropriate and is the work technically sound?

Yes

Reviewer Expertise:

Marine genomics

I confirm that I have read this submission and believe that I have an appropriate level of expertise to confirm that it is of an acceptable scientific standard.
